# Yoga for managing knee osteoarthritis in older women: a pilot randomized controlled trial

**DOI:** 10.1186/1472-6882-14-160

**Published:** 2014-05-18

**Authors:** Corjena Cheung, Jean F Wyman, Barbara Resnick, Kay Savik

**Affiliations:** 1 School of Nursing, University of Minnesota, Minneapolis, MN 55455, USA; 2 School of Nursing, University of Maryland, Baltimore, MD 21201, USA

**Keywords:** Yoga, Knee osteoarthritis, Symptom management, Older women

## Abstract

**Background:**

Osteoarthritis (OA) is a common problem in older women that is associated with pain and disabilities. Although yoga is recommended as an exercise intervention to manage arthritis, there is limited evidence documenting its effectiveness, with little known about its long term benefits. This study’s aims were to assess the feasibility and potential efficacy of a Hatha yoga exercise program in managing OA-related symptoms in older women with knee OA.

**Methods:**

Eligible participants (N = 36; mean age 72 years) were randomly assigned to 8-week yoga program involving group and home-based sessions or wait-list control. The yoga intervention program was developed by a group of yoga experts (N = 5). The primary outcome was the Western Ontario and McMaster Universities Osteoarthritis Index (WOMAC) total score that measures knee OA pain, stiffness, and function at 8 weeks. The secondary outcomes, physical function of the lower extremities, body mass index (BMI), quality of sleep (QOS), and quality of life (QOL), were measured using weight, height, the short physical performance battery (SPPB), the Pittsburgh Sleep Quality Index (PSQI), the Cantril Self-Anchoring Ladder, and the SF12v2 Health Survey. Data were collected at baseline, 4 weeks and 8 weeks, and 20 weeks.

**Results:**

The recruitment target was met, with study retention at 95%. Based on ANCOVAs, participants in the treatment group exhibited significantly greater improvement in WOMAC pain (adjusted means [SE]) (8.3 [.67], 5.8 [.67]; p = .01), stiffness (4.7 [.28], 3.4 [.28]; p = .002) and SPPB (repeated chair stands) (2.0 [.23], 2.8 [.23]; p = .03) at 8 weeks. Significant treatment and time effects were seen in WOMAC pain (7.0 [.46], 5.4 [.54]; p = .03), function (24.5 [1.8], 19.9 [1.6]; p = .01) and total scores (35.4 [2.3], 28.6 [2.1]; p = .01) from 4 to 20 weeks. Sleep disturbance was improved but the PSQI total score declined significantly at 20 weeks. Changes in BMI and QOL were not significant. No yoga related adverse events were observed.

**Conclusions:**

A weekly yoga program with home practice is feasible, acceptable, and safe for older women with knee OA, and shows therapeutic benefits.

**Trial registration:**

ClinicalTrials.gov: NCT01832155

## Background

Osteoarthritis (OA) is the most common form of arthritis that causes pain, functional limitation, and disability in older adults [1]. Prevalence was higher among women (25.4%) compared with men (17.6%), older age groups (50% for persons aged >65 years and 29.3% for persons aged 45--64 years) compared with younger age groups (7.9% for persons aged 18--44 years) [2]. The most commonly affected joints are the knees [1]. Symptomatic knee OA is associated with varying degrees of functional limitation, insomnia, [3] and reduced quality of life [4]. Because OA is a progressive and chronic degenerative condition that is highly prevalent, the health care utilization and costs for treatment are substantial. The total annual cost for medical treatments alone is estimated to be $81 billion [2]. As the population continues to age, the incidence of OA and its associated costs will continue to rise. No effective cure or disease-modifying treatments are currently available for OA; managing musculoskeletal pain and related symptoms are the most common reasons for people to seek non-traditional treatments [5].

Recent practice guidelines recognize that exercise programs such as aerobic and strengthening exercises are essential elements of any treatment program for OA [6]. Yoga is a mind-body practice in complementary medicine with origins in ancient Indian philosophy. The theory behind yoga practice is that the union of mind and spirit in exercise brings balance to the body and promotes healing [7]. Hatha yoga, the physical form of yoga, is commonly practiced in the United States [8]. It incorporates poses, breathing techniques, and meditation, can theoretically reduce pain and stiffness associated with OA by realigning the skeletal structure, strengthening muscles around the joints, and stretching tight joint structures [9]. The frequent joint motion when practicing yoga is believed to have physiologic effects at the cellular level. Because in vitro production of pro-inflammatory interleukin-1 zand tumor necrosis factor decreases under low-level intermittent fluid pressure, yoga exercise may reduce fluid pressure, which, in turn, preserves cartilage that would allegedly be lost by immobilization [10]. It is found to improve balance, strength, flexibility and relaxation in the general adult population [11], and have physiological benefits similar to those of moderate exercise [12]. Yoga is currently one of the fitness programs recommended by the Arthritis Foundation (AF) to promote joint flexibility and lower stress to potentially benefit individuals with arthritis in general [13]. Although yoga has been around for over 5,000 years and practiced worldwide over the past two decades, empirical evidence to validate its efficacy as an effective option for OA remains inconclusive.

Peer-reviewed, meta-analyses of yoga for musculoskeletal problems suggest that yoga is helpful for chronic pain and low back problems in younger adult population [14-16]; however, concerns of feasibility and safety remain when attempting to translate these studies to the older adult population. A literature review of yoga for arthritis supports its efficacy in reducing disease symptoms (tender/swollen joints, pain) and disability and improving self-efficacy and mental health [17]. However, most of the studies included in the review focus on rheumatoid arthritis (RA), only three were OA studies [18-20]. Because OA affects mostly older adults in their major weight-bearing joints, and RA more often affects a younger population and the smaller joints of their hands, wrists and feet, concerns of feasibility and safety remain when attempting to translate these studies to the OA population. Limited data are available to support the use of yoga for OA management. Three studies focused on yoga effects on knee OA with positive outcomes reported on symptoms including pain, flexibility, functional disability, anxiety, and quality of life [18-20]. Even though all three studies demonstrated positive effects of yoga on knee OA, these studies have methodological issues related to their designs. Two of the studies included small sample sizes (N ≤ 15), and the only randomized controlled trial conducted in India included participants whose mean age was 59 (35 to 80 years). Two of the three studies used Iyengar yoga as the intervention. Although Iyengar yoga is a style of Hatha yoga that is commonly practiced in the United States, the number of Iyengar teachers who are qualified to teach students with disability is limited. Additionally, none of these studies examined yoga acceptability and adherence which is essential for measuring long-term effects.

The purposes of this pilot study were to determine the feasibility, acceptability, and safety of an 8-week Hatha yoga program involving group and home-based sessions in community dwelling older women with knee OA, and to establish preliminary evidence for its effects on OA-related symptoms for a larger trial. Specifically, the study was aimed to: 1) examine the differences in pain, stiffness, physical function of the lower extremities (LE), quality of sleep (QOS), quality of life (QOL), and body mass index (BMI) between the treatment and wait-list control groups, 2) evaluate the effects of yoga on OA-related outcomes over time, 3) assess our ability to recruit and retain participants, and 4) measure adherence during yoga sessions and home practice.

## Methods

### Design

This pilot study used a randomized controlled trial (RTC) design with two arms. The treatment group received an 8-week Hatha yoga intervention involving group and home-based exercise sessions. The wait-list control group received the same yoga intervention after the treatment group completed the program at the end of 8 weeks. Participants in the wait-list control had a second baseline data collection at the end of the first eight weeks before participating in the yoga intervention. Participants were first assigned to an identification number based on the order of enrollment. They were then randomized by the principal investigator (PI) using simple randomization method. A sequence of computer-generated random numbers from 1 – 36 was used. Those who received an odd number were assigned to the treatment group and those who received an even number were assigned to the wait-list control group. Participants were informed of their group assignment by the PI. The research assistant who enrolled participants and collected the outcome data was blinded to the group assignment. Data were collected at baseline, 4 weeks, 8 weeks, and 20 weeks from the treatment group. Only baseline and 8 weeks data were collected from the wait list control group for between-group comparisons before they received the intervention. Once the wait list control group started the intervention program, the same data collection protocol applied. The 20 weeks follow-up outcome was used to evaluate the carry-over effects of the yoga intervention and post-intervention exercise adherence. Research incentives of $10 were given at each data collection point to participants in both groups. In addition, each participant was given a yoga mat for use in group-based classes and home practices. The research protocol was approved by the St. Catherine University and University of Minnesota Institutional Review Boards. Informed consents were obtained from all participants involved in the study.

### Sample and setting

**Figure 1:**
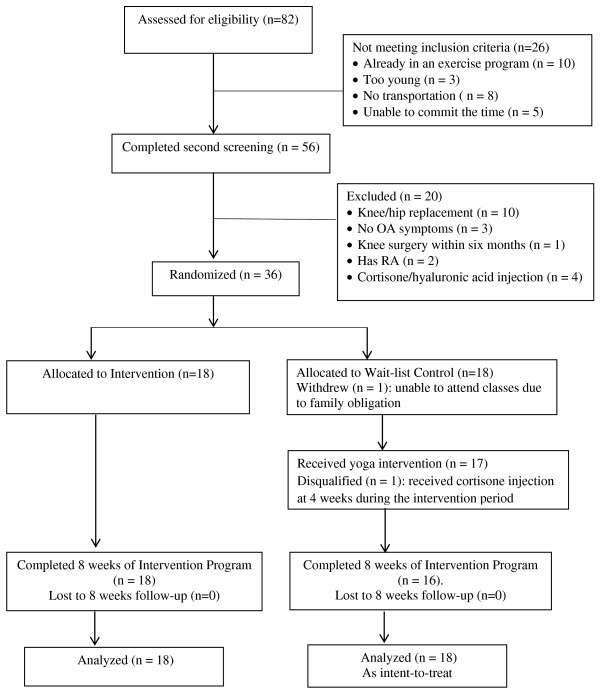
CONSORT Flow diagram of study participants.

Thirty-six community-dwelling women were recruited and randomized (Figure 1). Recruitment took place from February 2011 – September 2011 through placing flyers in various senior centers; distributing press release to the University’s Alumnae Monthly Newsletter, local and senior newspapers, accessing the data base and mailing invitation letters out to patients meeting demographic and diagnostic criteria from the University of Minnesota Physician Practice. Potential participants who provided their contact information or called the study line were screened via telephone by a trained research assistant for eligibility which was based on the following inclusion criteria: (a) community-dwelling women between the ages of 65 and 90 years; (b) had a symptomatic OA of knee diagnosis for at least 6 months; (c) had no previous training in any form of yoga; and (d) were not currently participating in a supervised exercise program. Once initial screening eligibility was conducted, the individual was further screened at a location that was convenient to the participant to confirm the presence of knee OA symptoms using the Clinical Criteria for the Classification of Idiopathic OA of the Knee developed by the American College of Rheumatology [21] which does not require any radiographic evidence [22]. Potential participants’ cognitive ability was assessed by using the Short Portable Mental Status Questionnaire (SPMSQ) [23]. Individuals were excluded if the score was less than eight which indicates moderate/severe cognitive impairment. It was at this time that the individual was evaluated for the following exclusion criteria: symptoms of joint locking; instability indicated by chronic use of a knee brace, cane, walker, or wheelchair; a corticosteroid injection in the symptomatic joint within three months of study entry; a hyaluronic acid injection in the symptomatic joint within six months of study entry; a history of knee surgery within the last two years or a joint replacement at any point. Individuals who had self-reported significant medical comorbidities that might preclude exercise participation such as: a) uncontrolled high blood pressure or existing heart condition; and b) other comorbid condition with overlapping symptoms (i.e. fibromyalgia, rheumatoid arthritis) were also be excluded. Individuals who had medication changes for arthritis symptoms were permitted to remain in the trial; however, these changes were monitored. This pilot study did not perform a formal power calculation. The sample size of 36 was determined based on recruitment feasibility over the time frame for this study; not on providing adequate power to detect expected between group differences in mean efficacy outcomes. All the intervention classes were held at a yoga studio which was conveniently located near a city bus stop and had sufficient parking spaces.

### Intervention

The yoga program was composed of one 60-minute Hatha yoga class per week for eight weeks. Sessions included *asanas* (poses) in the seated, supine, and standing positions; *pranas* (breathing); and meditation. A progressive series of poses was used with static stretching, balance, and strength exercises. Classes were designed by a panel of five certified/registered yoga teachers who had experiences teaching older adults. The program was reviewed by two yoga researchers and a yoga master. The yoga intervention program was specifically for older adults with knee OA; the class size was kept small with nine participants per class. Because yoga postures are highly modifiable, props such as yoga mat, blocks, strap, blankets, and chair were used during class, and poses were modified when needed based on the participants’ physical limitations. Key yoga postures used in the program included mountain pose, warrior I and 2 poses, tree pose, chair pose, easy seated pose, bound angle poses, open angle pose, half locust variation, bridge pose, standing forward fold, reclining hamstring stretch, reclining twist, and relaxation pose. All classes were taught by the same registered yoga teacher who had over 10 years of yoga teaching experience. In addition to attending classes, participants were instructed to practice 30-minute yoga four times a week at home. Researcher developed handouts of yoga exercises illustrating poses included in each weekly program were distributed at the end of each session to help participants practice the poses correctly at home. The study followed the intervention protocol that was developed by the research team. Study quality was monitored by two established research mentors who served as the Data Safety and Monitoring Board (DSMB) members. Progress reports including participant recruitment, retention/attrition with reasons for dropout from the study, and a summary of adverse events were sent to DSMB members for independent reviews. Quarterly meetings were held between the research mentors and the PI to review the study progress.

### Measures

Primary outcome measures included: OA symptoms (e.g., pain, stiffness and physical function) were assessed using the Western Ontario and McMaster Universities OA Index scale (LK scale 3.1) (WOMAC) [24] and a single question that asked about the number of pain medications (prescription or over the counter analgesics) used per day. Secondary outcome measures included physical performance of the LE which was assessed using the Short Physical Performance Battery (SPPB) developed by the National Institute on Aging [25]. The test consists of three components: repeated chair stands, balance, and timed 8 foot walk. BMI was calculated using the participant’s weight and height. QOS was evaluated using the Pittsburgh Sleep Quality Index (PSQI) [26]. A score of ≥ 5 on the PSQI total scale, which is computed as a sum of the seven subscales (e.g., sleep quality, sleep latency, sleep duration, sleep disturbance, sleep efficiency, and use sleep medication) is associated with clinically significant sleep disruptions, including insomnia and major mood disorders. The self-perceived QOL was assessed using the Short Form Health Survey; the SF-12 which measures both physical and mental component summary scales [27] and the Cantril Self-Anchoring Ladder that measures both “current” and “in 5 years” [28].

Feasibility measures: Feasibility was measured by eligibility, recruitment, and retention rates. Reasons for not participating and for withdrawing from the study were collected. Acceptability was evaluated by the participants’ ratings of perceived difficulty of the yoga class, level of enjoyment, whether they would recommend the class to others with OA, as well as their exercise adherence during and after the yoga program. Perceived level of difficulty and enjoyment were rated by participants after completing the yoga program using a scale of 1–10 where 10 represents extremely difficult and most enjoyable, respectively. Exercise adherence was evaluated by the percentage of yoga sessions attended and percentage and number of days yoga was practiced at home as self-reported by the participant on a log sheet. Safety during group sessions was monitored by either the PI or research staff, one of them was present in all yoga classes. Safety during home yoga practice was monitored by the participants themselves. They were asked to record the type and severity of any injuries that occurred during their home practice on a log sheet and bring it to class each week. Demographic information (e.g., age, race/ethnic background, education level, annual household income, marital status, living arrangement, and type of insurance), BMI, and comorbidities were collected from all participants.

### Procedures for data collection and safety

Both primary and secondary measures from the treatment group were collected at baseline, 4 weeks, 8 weeks, and 20 weeks. Participants in the wait list control were instructed to carry on their usual care for 8 weeks. Their outcome variables were assessed at baseline and 8 weeks after randomization into the trial. The 8 weeks data from the wait list control group also served as the second baseline before their intervention began. Once the wait list control group started receiving the intervention, additional data were collected at 4 weeks, 8 weeks, and 20 weeks. All feasibility measures were collected throughout the intervention program and at 20 weeks follow-up. During intervention, a log sheet was provided for self-recording the frequency and specific yoga pose practiced as well as any adverse events that occurred during yoga practices at home such as injury or worsening of OA symptoms. The identification number from 1 – 36 that participants received upon enrollment was used on their log sheet to ensure anonymity facilitating honest reporting of their exercise adherence.

### Statistical analysis

Descriptive statistics including means, standard deviations, ranges, and frequency distributions were used to describe the profiles of the participants from demographic data gathered, the acceptability, and safety of the yoga program. An independent t-test was used to test significant differences in demographic and baseline outcome variables between the treatment and control group at baseline. This study was analyzed in standard procedure for a wait list control group design. An intent-to-treat analysis approach was used. First, the main aim was analyzed comparing the treatment and wait list control group after the end of the first 8 weeks. An analysis of covariance (ANCOVA), using the baseline score as the covariate, was used to test differences in outcome measures between treatment and control groups upon the completion of the intervention program after the first eight weeks. Second, the first baseline and second baseline in the control group were compared using a paired t-test to see if any significant changes had occurred over the 8 weeks prior to beginning the yoga intervention. Any differences would be controlled for as the baseline would be used as a covariate in the subsequent analysis. Combining data from both treatment and wait-list controls into one group (both post-treatment) with baseline variables as the covariate, a repeated measures ANCOVA model, using mixed models, was employed to assess changes over time in primary and secondary outcome measures. An intent-to-treat analysis approach was used by analyzing with mixed models. This is the recommended modeling for intent-to-treat analyses. Using the data as is within a mixed model analysis has a lower type I error and higher power than any type of imputation method used for missing data, which would be needed for a repeated measures ANCOVA. Also, imputation may result in biased estimates of effects and standard errors [29]. Alpha levels for the multiple time comparisons were controlled for using Tukey’s LSD which computes the least difference between the possible pairwise comparisons that can be considered significant. Data were analyzed using the Statistical Package for the Social Science (SPSS) Version 18.0. If the calculated *p-* value was equal or less than .05, the results were considered statistically significant.

## Results

A total of 82 potential participants were screened. Fifty-six of them met the inclusion criteria and were eligible for second screening. Recruitment took nine months. Thirty-six community- dwelling older women with knee OA who met all the study criteria and were able to commit to the duration of the yoga intervention program were enrolled. The remaining potential participants were excluded for a variety of reasons, the major ones being hip/knee replacement or already in an exercise program (Figure 1). Data were collected at four time points (baseline, 4 weeks during treatment, 8 weeks, and 20 weeks) and the trial was ended after the follow-up data were collected from both treatment and wait list control groups.

### Baseline data

The participants had a mean age of 72 years and mean BMI of 29 kg/m^2^. They were predominately white (86%). Demographic characteristics and baseline outcomes differences between the treatment and control group are listed in Table 1. Participants in treatment group had significantly lower level of education (*p* = .01) and higher number of co-morbidities (*p* = .03).

**Table 1:** Baseline characteristics of the study participants

	**Yoga**	**Wait list control**	
**Variable**	**Mean (95% CI)**	**Mean (95% CI)**	***p*-value**
**Demographics**			
Age, years	71.9 (69.3, 74.6)	71.9 (69.0, 75.0)	1.0
BMI	29.1 (26.7, 31.7)	28.8 (26.0, 31.7)	.80
Education, years	15.7 (14.1, 16.3)	17.0 (16.1, 18.0)	.01
**Disease condition (range of scores)**			
WOMAC-pain (0–20)	9.3 (7.4, 11.3)	7.7 (5.6, 9.8)	.25
WOMAC-stiffness (0–8)	5.2 (4.5, 5.9)	4.3 (3.3, 5.3)	.16
WOMAC- physical function (0–68)	35.0 (29.2, 40.9)	27.1 (19.6, 34.6)	.09
WOMAC-total (0– 96)	49.5 (41.6, 57.4)	39.2 (29.7, 48.7)	.09
SPPB Global (0–12)	9.2 (8.1, 10.3)	8.8 (7.6, 10.0)	.57
SPPB-chair (0–4)	2.4 (1.7, 3.1)	1.9 (1.2, 2.7)	.36
SPPB-balance (0–4)	3.6 (3.1, 4.0)	3.7 (3.3, 4.1)	.59
SPPB-walk (0–4)	3.3 (3.0, 3.6)	3.1 (2.7, 3.5)	.49
Comorbidities, no.	2.8 (1.7, 3.9)	1.4 (.8, 2.0)	.03
**QOS and QOL (range of scores)**			
PSQI Total (0–21)	6.5 (4.4, 8.6)	5.7 (3.9, 7.4)	.52
PSQI-sleep efficiency (0–3)	.61 (.1, 1.2)	.61 (.15, 1.2)	1.0
PSQI-sleep disturbance (0–3)	1.7 (1.5, 2.0)	1.5 (1.2, 1.8)	.18
PSQI-sleep duration (0–3)	.44 (.1, .8)	.33 (.05,.51)	.65
PSQI-sleep latency (0–3)	1.2 (.7, 1.7)	.76 (.6, 1.5)	.23
PSQI-sleep quality (0–3)	1.1 (.7, 1.4)	.83 (.4, 1.4)	.38
PSQI-Use sleep medications (0–3)	.67 (.1, 1.3)	.78 (.2, 1.4)	.78
SF-12 PCS (0–100)	39.5 (36.4, 42.6)	37.8 (35.3, 40.3)	.37
SF-12 MCS (0–100)	51.0 (48.2, 53.8)	51.7 (48.8, 54.6)	.72
Cantril Current (0–10)	6.9 (6.0, 7.8)	7.8 (7.1, 8.5)	.11
Cantril 5 years (0–10)	7.3 (6.5, 8.1)	7.8 (7.0, 8.5)	.41

n = 18 per group. WOMAC = Western Ontario and McMaster Universities Osteoarthritis Index (lower scores = better state); SPPB = Short Physical Performance Battery (higher scores = better state); QOS = quality of sleep; QOL = quality of life; PSQI = Pittsburgh Sleep Quality Index (lower scores = better state); SF-12 = Health related Short Form 12 (higher scores = better state); PCS = physical component summary; MCS = mental component summary; Cantril current and 5 years (higher scores = better state); *P* values ≤ .05.

### Effects of Yoga on OA related symptoms at 8 weeks

The 8-week measures for the all scores in between-group analysis with baseline as covariate are shown in Table 2. Although the study was not powered to detect significant changes in outcome, the between-group differences between treatment and control groups at 8 weeks were significant for WOMAC pain (adjusted mean [SE]) (8.3 [.67], 5.8 [.67]; *p* = .01) and stiffness index scores (4.7 [.28], 3.4 [.28]; *p* = .002 with a trend for significance for the total score (39.3 [3.0], 31.0 [3.0]; *p* = .06). The treatment group compared to the control group improved significantly for the SPPB-repeated chair stands subscale (adjusted mean [SE]) (2.0 [.23], 2.8 [.23]; p = .03). None of the other differences in outcome measures (PSQI, SF-12 or Cantril’s ladder) reached significance at 8 weeks.

**Table 2:** Comparison between Yoga and control groups at 8 weeks

	**Control (n = 18)**		**Yoga (n = 18)**		**Difference**	
	**Baseline mean(SD)**	**8 weeks adjusted mean(SE)**	**Baseline mean(SD)**	**8 weeks adjusted mean(SE)**	**At 8 weeks mean(SE)**	**p-value**^**1**^
**WOMAC**						
Total	39.2(19.1)	39.3(3.0)	49.5(15.8)	31.0(3.0)	8.3(4.3)	.06
Pain	7.7(4.2)	8.3(.67)	9.3(4.0)	5.8(.67)	2.5(.96)	.01
Stiffness	4.3(2.0)	4.7(.28)	5.2(1.4)	3.4(.28)	1.3(.41)	.002
Function	27.1(15.2)	26.2(2.3)	35.0(11.8)	22.0(2.3)	4.2(3.3)	.21
**SPPB**						
Global	8.4(3.1)	9.0(.37)	9.2(2.2)	10.0(.37)	−1.0(.52)	.06
Repeated chair stands	2.1(1.4)	2.0(.23)	2.4(1.4)	2.8(.23)	-.78(.37)	.03
Balance	3.3(1.1)	3.8(.13)	3.6(.98)	3.8(.13)	-.04(.19)	.83
8″ Walk	3.0(1.1)	3.1(.16)	3.3(.67)	3.5(.16)	-.75(.32)	.16
**PSQI**						
PSQI Total	5.4(2.8)	6.1(.52)	6.5(4.2)	5.0(.52)	1.1(.73)	.15
Sleep quality	.89(.96)	.90(.13)	1.1(.64)	.66(.13)	.24(.18)	.20
Sleep latency	1.0(.91)	.81(.15)	1.2(.99)	1.1(.15)	-.25(.21)	.25
Sleep duration	.28(.46)	.39(.15)	.44(.71)	.16(.15)	.23(.21)	.29
Sleep disturbance	1.4(.51)	1.5(.15)	1.7(.46)	1.4(.15)	.18(.21)	.41
Use sleep meds	.61(1.1)	.80(.19)	.67(1.2)	.70(.19)	.10(.26)	.70
Sleep efficiency	.67(1.0)	.60(.18)	.61(1.1)	.45(.18)	.15(.26)	.56
**SF-12**						
MCS	53.4(4.8)	51.7(1.2)	51.0(5.7)	49.7(1.2)	1.5(1.7)	.39
PCS	33.9(4.4)	38.7(1.0)	39.5(6.2)	38.0(.98)	.69(1.5)	.65
**Cantril ladder**						
Current	7.3(1.3)	7.7(.37)	6.9(7.8)	7.5(.37)	.15(.52)	.78
In 5 years	6.9(2.2)	7.8(.32)	7.3(1.6)	7.2(.32)	.64(.45)	.16
**BMI**	28.9(5.8)	28.9(.16)	29.2(5.0)	28.7(.16)	.21(.22)	.36

^1^ANCOVA comparing 8 week results with 8 weeks means adjusted for baseline, adjusted mean (SE) reported. WOMAC = Western Ontario and McMaster Universities Osteoarthritis Index; SPPB = Short Physical Performance Battery; PSQI = Pittsburgh Sleep Quality Index; SF-12 = Health related Short Form 12; PCS = physical component summary; MCS = mental component summary; Cantril Ladder (quality of life scale).

### Effects of Yoga on OA related symptoms over time

As there were no significant differences between the first and second baseline scores in the wait-list control group except for SF-12 PCS, data for all participants from both treatment and wait-list control groups (both post-treatment) were combined (N = 36) for repeated measures analyses to increase the precision of estimates for differences (Table 3). From 4 weeks to 8 weeks during intervention, both WOMAC pain and total scores improved significantly (adjusted mean [SE]) (7.0 [.49], 5.9 [.5], p = .04) and (35.3 [2.1], 30.9 [2.2], p = .05) and the effect sustained at 20 weeks follow-up (5.4 [.5], p = .01) and (28.5 [2.2]), respectively. WOMAC function improved from 4 weeks to 20 weeks (24.4 [1.6], 19.8 [1.7]; p = .008). Changes in the number of analgesics (both prescription and over the counter oral analgesics) taken for knee OA was not significantly different throughout all time points.

**Table 3:** Effects of yoga intervention on OA related symptoms at different time points (N = 36)

	**Time 1 (T1) 4 weeks**	**Time 2 (T2) 8 weeks**	**Time 3 (T3) 20 weeks**	**p-value**^**1 **^**T1 vs. T2**	**p-value**^**1 **^**T1 vs. T3**	**p-value**^**1 **^**T2 vs. T3**
**WOMAC**						
Pain	7.0 (.49)	5.9 (.50)	5.4 (.50)	.04	.01	.38
Stiffness	3.8 (.22)	3.6 (.22)	3.4 (.22)	.47	.14	.31
Function	24.4 (1.6)	21.4 (1.7)	19.8 (1.7)	.06	.008	.31
Total	35.3 (2.1)	30.9 (2.2)	28.5 (2.2)	.046	.007	.27
Use pain meds	.64 (.13)	.76 (.13)	.79 (.13)	.47	.35	.86
**SPPB**						
Repeated chair stands	2.5 (.19)	2.8 (.19)	2.8 (.19)	.08	.16	.98
Balance	3.7 (.09)	3.9 (.09)	3.7 (.09)	.13	.78	.20
Walk	3.4 (.11)	3.6 (.11)	3.4 (.11)	.03	.58	.11
Global	9.6 (.29)	10.2 (.29)	9.9 (.29)	.007	.34	.10
**PSQI**						
PSQI total	5.5 (.31)	5.1 (.32)	6.8 (.32)	.28	<.001	<.001
Sleep quality	.87 (.10)	.70 (.10)	.73 (.10)	.08	.12	.77
Sleep efficiency	.54 (.13)	.39 (.13)	.42 (.13)	.20	.33	.81
Sleep disturbance	1.6 (.08)	1.4 (.09)	1.3 (.09)	.11	.01	.53
Sleep latency	.92 (.11)	.88 (.11)	.88 (.11)	.73	.74	.99
Sleep duration	.41 (.08)	.23 (.09)	.35 (.08)	.15	.33	.34
**SF-12**						
PCS	37.4 (.77)	37.4 (.78)	37.7 (.78)	.94	.75	.70
MCS	50.0 (.82)	51.2 (.83)	50.5 (.84)	.23	.63	.52
**Cantril Ladder**						
Current	6.9 (.24)	7.4 (.24)	7.3 (.24)	.045	.19	.58
In 5 years	7.3 (.21)	7.3 (.21)	7.2 (.21)	.92	.61	.52

Adjusted Means (SE).

^1^Repeated measures ANOVA with overall alpha controlled by use of Tukeys LSD for multiple comparisons. WOMAC = Western Ontario and McMaster Universities Osteoarthritis Index; SPPB = Short Physical Performance Battery; PSQI = Pittsburgh Sleep Quality Index; SF-12 = Health related Short Form 12; PCS = physical component summary; MCS = mental component summary; Cantril Ladder (quality of life scale).

Notably, significant improvements were seen in SPPB 8 foot walk from 4 to 8 weeks (adjusted mean [SE]) (3.4 [.11], 3.6 [.11]; p = .03) and global scores (9.6 [.29], 10.2 [.29]; p = .007). However, scores in balance test at each assessment point did not appear to show any clinically noticeable change. Sleep disturbance was significantly improved from 4 weeks during intervention to 20 weeks follow-up (adjusted mean [SE]) (1.6 [.08], 1.3 [.09]; p = .01). In contrast, PSQI total scores were significantly worse from 4 and 8 weeks to 20 weeks follow-up. No significant improvement was noted in SF12 over time but remained steady throughout all time points. The Cantril Self-Anchoring Ladder “QOL current” scores significantly improved from 4 weeks to 8 weeks (adjusted mean [SE]) (6.9 [.24], 7.4 [.24]; p = .045). For “QOL in 5 years,” the changes were not significant.

### Feasibility outcomes

The study dropout rate was 5%. One participant from the control group dropped out after the second screening due to her schedule not allowing her to attend the class session and another participant dropped out after four weeks of intervention due to severe knee pain affecting her mobility.

The majority of participants (n = 25) attended ≥ 75% of classes, with common barriers included being too busy or illness. Only 33% of participants practiced yoga at home as prescribed (30 minutes a day, four days a week) during the active intervention period. The average duration of yoga practice was 112 minutes out of 120 minutes per week. Seventy percent of participants practiced yoga at home ≥ 4 days/week, but only 36% of them practiced 30 minutes each time. At 20 weeks follow up, 74% of participants reported still practicing yoga at home for OA management. A majority of them (55%) practiced 1–2 days and 45% practiced 3–4 days of yoga per week. Twenty percent of participants practiced 5–10 minutes, 40% practiced 11–20 minutes, and another 40% practiced 21–30 minutes per day of yoga at home. None practiced 5 times per week and/or 30 minutes per day as recommended.

### Acceptability and safety

Upon completion of the yoga program, participants reported the yoga intervention program was enjoyable. The average score was 9 (ranges from 7 to 10) on a scale of 0–10, where 10 represents extremely enjoyable. The average rating of the difficulty level of the yoga program was 4 (ranges from 0 to 9) on a scale of 0 – 10, where 10 represents extremely difficult. All participants expressed that they would recommend the yoga program to others with OA. Participants did not report any yoga practice related adverse events/injuries although one participant decided to receive cortisone injection. She was removed from the study once this occurred as she was no longer eligible for participation.

## Discussion

The major findings of this pilot study were that this 8-week Hatha yoga program was safe, feasible, and acceptable for older women with knee OA. This study provides important information regarding recruitment, retention, intervention and post-intervention effects, and frequency of yoga practice that were achieved among older women with knee OA in an 8 week intervention. After the 8-week Hatha yoga program, participants experienced significant reductions in OA symptoms and increases in some physical function of the LE with the sizes of these effects ranging from small (.37 and .41) for the SPPB (repeated chair stands) test and WOMAC stiffness measure to large (.96) for the WOMAC pain measure, which were similar to results from a previous study [18]. There was also a trend for significant improvement in the WOMAC total and SPPB global scores. Even though other comparisons did not reach significance, the effect sizes for the differences in these measures will be used to plan future studies.

When data from both treatment and the wait-list control groups were combined in repeated measures, the SPPB global score and the WOMAC total score were significantly improved at 8 weeks and 20 weeks follow up respectively. The sustainment of improvements in WOMAC measures at the 20 weeks follow up occurred even with the decreased frequency of yoga practice after the intervention period. However, because none of the participants reported joining any new yoga classes after the intervention program and most participants did not adhere to the frequency of yoga practice at home, it is not surprising that the effects of yoga on physical function of the LE did not sustain at 20 weeks follow up. The American College of Sports Medicine recommends that older adults take part in either moderate aerobic exercise for 30 minutes per day, five times per week or vigorous aerobic exercise for 20 minutes per day, three times per week for maintaining musculoskeletal fitness [30].

The significantly worse PSQI total scores at 20 weeks follow up compared to both time points during yoga intervention deserves further investigation. Factors associated with older women with knee OA that may contribute to poor QOS, timing and dose of yoga needed for promoting sleep quality, and strategies to enhance yoga adherence will need to be investigated.

The Cantril Self-Anchoring Ladder current QOL measure was perceived to be improved significantly after 8 weeks of yoga intervention compared to the control group, but the improvement did not differ significantly in the 5 years (future) QOL or the SF 12 measures at any time points. In general, independent living older adults who volunteered to be in a research study may have a positive outlook in life [31]. The Cantril Self-Anchoring Ladder scores were high at baseline before the intervention began; therefore, ceiling effects might have contributed to the findings.

The methodological quality used in this study was higher than in most prior yoga research because we used a RCT design and an assessor was blinded to group allocation and data entry to reduce the risk of bias. The inclusion and exclusion criteria were well defined, and the yoga intervention program that was specifically designed by a group of yoga experts for older women with knee OA can be easily replicable by other qualified yoga teachers. The poses are gentle and adaptable to accommodate the needs and limitations of older women who have functional imitations of the LE due to knee OA. Additionally, the study participants were older women recruited from a general community setting with various levels of OA symptoms and medical histories, demonstrating the ability of the yoga program to meet the needs of older women with a range of abilities and comorbidities. Although there were minor, temporary musculoskeletal soreness and pains experienced by a small number of individuals, no yoga related adverse effects were detected during the intervention classes and home practices.

High attrition rates have been reported among older exercisers in general [32]. This 8-week Hatha yoga intervention program exhibited a low attrition rate (5%). The feasibility of the yoga program was also demonstrated by the excellent attendance with the majority of participants attending 75% or more of the group classes. This indicates that the program was acceptable to the older participants with knee OA and also relatively easy to learn and enjoyable as reported by the participants. Low rates of exercise adherence among older adults with musculoskeletal conditions clearly warrant the need for evidence-based programs that are acceptable and enjoyable.

Despite the excellent short term yoga adherence rate, none adhered to the recommended frequency (number of days/week or minutes/day) of home yoga practice at 20 weeks follow-up even though many participants reported still practicing yoga on a regular basis. The issues associated with group versus individual exercise are critical for many people [33]. Home-based exercise program will remain an integral part of health care management in this population. Additional research is needed to determine effective strategies to improve long term home-based exercise adherence in older adults with OA, and the optimal dose of home yoga practice for sustaining therapeutic effects.

There are several study limitations. The use of a wait-list control group with the extended time commitment prior to intervention may have affected study participation; however, it does offer the advantages of obtaining information on the long-term adherence and maintenance of outcomes in a larger sample than has previously been possible. Although the research staff was not informed of the group assignment, the data collection schedule (data at 4 weeks were collected only from participants who were receiving treatment) did not allow for blinding to be maintained. Another limitation was the small sample size limiting generalizability. Results from this pilot study will lead to a better-designed main study and serve as a discussion point about setting a threshold value for calculating the sample size. Our study has several notable strengths including the use of an expert panel of yoga teachers to design a program specifically for older women with OA, a RCT design, data collection at multiple time points, and the length of the post-intervention follow up period.

## Conclusions

A weekly group-based yoga program with home practice appears to be a feasible and safe option for older women with knee OA that may lead to improvements in symptoms and physical function of LE, but is inconclusive for QOS and QOL. The Osteoarthritis Research Society International recommended that optimal management of OA requires a combination of non-pharmacological and pharmacological modalities and that the initial focus should be on self-help and patient-driven treatments rather than on passive therapies delivered by health professionals [34]. Yoga is safe and acceptable to be practiced by older women with OA. Future research is needed to determine the timing and dose response of yoga practice and outcomes.

## Abbreviations

AF: Arthritis foundation; ANCOVA: Analysis of covariance; BMI: Body mass index; LE: Lower extremities; OA: Osteoarthritis; PQSI: Pittsburgh sleep quality index; QOL: Quality of life; QOS: Quality of sleep; SF12: Short form health survey; SPMSQ: Short portable mental status questionnaire; SPPB: Short physical performance battery; SPSS: Statistical package for the social science; WOMAC: Western Ontario and McMaster Universities Osteoarthritis Index.

## Competing interests

The authors declare that they have no competing financial or non-financial interests.

## Authors’ contributions

CC conceived the study, carried out the design, interpretation of results, and writing the manuscript. JW participated in the design of the study, assisted with recruitment, and critically reviewed and revised the manuscript. BR participated in the design of the study and critically reviewed and revised the manuscript. KS performed all the statistical analyses and helped drafted the manuscript. All authors read and approved the final manuscript.

## Authors’ information

CC, JW and KS are faculty/staff at the University of Minnesota, School of Nursing where CC is an Assistant Professor, JW is a Professor, and KS is a Biostatistician. BR is a Professor at the University of Maryland, School of Nursing.

## Pre-publication history

The pre-publication history for this paper can be accessed here:

http://www.biomedcentral.com/1472-6882/14/160/prepub
